# Investigation of cerebral cortical morphological similarity and network topological abnormalities in hepatic encephalopathy utilizing a morphometric inverse divergence network framework

**DOI:** 10.3389/fneur.2026.1830519

**Published:** 2026-07-06

**Authors:** Chengkun Hong, Taipeng Zeng, Xiaoyang Wang, Li Chen, Minghui Mao, Hao Huang, Jianfeng Chu, Liyuan Fu

**Affiliations:** 1 Fuzong Teaching Hospital of Fujian University of Traditional Chinese Medicine (900th Hospital), Fuzhou, Fujian, China; 2 College of Integrative Medicine, Fujian University of Traditional Chinese Medicine, Fuzhou, Fujian, China; 3 Academy of Integrative Medicine, Fujian University of Traditional Chinese Medicine, Fuzhou, Fujian, China; 4 Department of Hepatobiliary Disease, Fuzong Clinical Medical College of Fujian Medical University, Fuzhou, Fujian, China

**Keywords:** graph theory, hepatic encephalopathy, magnetic resonance imaging, morphological similarity, morphometric inverse divergence

## Abstract

**Background:**

Hepatic encephalopathy (HE) requires objective biomarkers for early diagnosis and mechanistic clarification. This study first integrates the Morphometric Inverse Divergence (MIND) network with graph theory to explore cerebral cortical morphological similarity and topological abnormalities in HE, cirrhotic non-HE (NHE), and healthy control (HC) groups.

**Methods:**

A total of 31 HE, 30 NHE patients and 30 HCs were enrolled for 3.0T magnetic resonance imaging (MRI) 3D-T1WI scanning. FreeSurfer was used for image preprocessing, and 5 cortical morphological features were extracted based on the Schaefer-400 atlas. MIND networks were constructed via symmetric Kullback–Leibler divergence, graph theory was applied to extract topological properties, and intergroup differences were analyzed by general linear model (GLM).

**Results:**

Compared with HCs, HE patients exhibited significantly elevated mean MIND values across multiple functional subnetworks, including the visual (VIS; *t* = 3.629, *p* = 0.004), default mode (DMN; *t* = 3.115, *p* = 0.009), limbic (LMB; *t* = 2.969, *p* = 0.009), frontoparietal (FPN; *t* = 2.917, *p* = 0.009), and ventral attention (VAN; *t* = 2.212, *p* = 0.043) networks. Graph theoretical analysis revealed increased global efficiency (Eglob, *t* = 2.681, *p* = 0.0100) and local efficiency (Eloc, *t* = 2.683, *p* = 0.010). NHE patients showed mild DMN connectivity enhancement in edge analysis, but no significant differences in subnetwork mean MIND and nodal metrics (all *p* > 0.05), exhibiting a non-significant transitional trend in efficiency indices between HE and HC groups.

**Conclusion:**

HE causes abnormal whole-brain cortical morphological covariance networks with enhanced connectivity and efficiency, and NHE has early-stage network alterations. MIND network indices are potential imaging biomarkers for HE diagnosis and monitoring, supplementing the neuropathological mechanism of HE and making up for the limitations of traditional structural covariance network research in HE.

## 1 Introduction

Hepatic encephalopathy (HE) is a syndrome of central nervous system dysfunction characterized by metabolic disturbances, resulting from acute or chronic severe hepatic insufficiency or portosystemic shunting (1, 2). Patients with liver cirrhosis non-HE (NHE), who have not yet developed typical neuropsychiatric symptoms but may already exhibit early neurobiological alterations associated with the occurrence and progression of HE, represent an ideal observational window for exploring the pathological mechanisms of HE (3), and their research value is particularly critical. The specific neuropathological mechanisms underlying HE remain incompletely elucidated, and the key to achieving its early diagnosis and intervention lies in the development of objective and sensitive neuroimaging biomarkers.

Neuroimaging studies have confirmed the presence of extensive cerebral structural abnormalities in patients with HE (4). For instance, voxel-based morphometry (VBM) and cortical thickness measurements have revealed widespread, symmetrical alterations in gray matter volume among HE patients. Specifically, increased gray matter volume was observed in certain regions such as bilateral thalamus, lingual gyrus, calcarine fissure and surrounding cortex, whereas decreased gray matter volume was found in other regions including bilateral insula, basal ganglia, anterior cingulate cortex, and cerebellum (5, 6). Traditionally, research on brain structural abnormalities has primarily focused on measuring local volume or cortical thickness in specific brain regions (7–11). However, the brain is a highly integrated, complex network system. Individual brain regions do not change independently during development, aging, or disease processes; instead, they often exhibit coordinated structural alterations, known as morphological similarity or covariance. Capturing these whole-brain, systematic patterns of morphological covariation is crucial for gaining a deeper understanding of the neuropathological mechanisms underlying disorders such as HE. To investigate this type of whole-brain network co-variation, the Morphometric Similarity Networks (MSNs) is constructed by calculating correlations between vectors of multiple morphological features across brain regions, providing a powerful tool for studying such issues (12–15). However, traditional MSN analysis methods possess certain limitations: the summarization of vertex-level features into regional means results in loss of information regarding subtle structural heterogeneity within brain regions, and the processes of feature extraction and network construction are susceptible to influences from image resolution, registration errors, and variations in scanning protocols, thereby limiting cross-study stability and comparability (16–19).

To address the aforementioned research gaps, this study employs Morphometric Inverse Divergence (MIND) to construct and analyze cortical similarity networks. MIND represents an emerging and more reliable method for constructing MSNs (20–22). Unlike MSNs, which aggregate vertex-level data into regional summary statistics, MIND integrates multiple structural features (e.g., cortical thickness, surface area, curvature) at the vertex level and quantifies inter-regional similarity by computing the symmetric Kullback–Leibler (KL) divergence between the multivariate distributions of these morphological features (23). Research by Sebenius et al. (24) demonstrates that MIND networks exhibit higher test–retest reliability, greater consistency with cortical cytoarchitectonics and tract-tracing measures of axonal connectivity, and increased sensitivity to age-related changes, indicating a stronger biological and genetic foundation. The MIND methodology has already demonstrated significant value in research on psychiatric disorders such as schizophrenia and depression (25–27), however, its application in the study of HE remains unexplored.

The organizational properties of brain networks can be quantified using graph theory. Graph theory provides a suite of topological metrics, encompassing both global and nodal levels (28–30), These metrics collectively delineate the organizational principles of the brain as a complex system, effectively characterizing the efficiency with which brain networks integrate and segregate information. Concurrently, whether cortical MSNs constructed using the MIND method exhibit topological abnormalities in HE constitutes a completely uncharted area of research.

Therefore, this study is the first to apply the MIND method to construct whole-brain cortical MSNs and, combined with graph theory analysis, systematically compare the differences in these networks among the HE group, the NHE group, and healthy controls (HC). We hypothesize that: (1) Compared to HC, both HE and NHE patients will exhibit altered connection strengths in their MIND networks; (2) HE patients will demonstrate disrupted MIND network topology, manifesting as a pathological topological reorganization rather than a complete shift toward random architecture. This study aims to elucidate the neurostructural basis of HE from the novel perspective of topological disorganization in cortical morphological covariance networks, potentially providing neuroimaging biomarkers for its early diagnosis.

## 2 Materials and methods

### 2.1 Research design and subjects

This study employed a prospective cross-sectional design and enrolled a total of 61 cirrhotic patients who met the diagnostic criteria outlined in the *Evidence-Based Clinical Practice Guidelines for Cirrhosis* (31), alongside 30 HC. The definitions of the patient groups were strictly delineated based on the West Haven criteria (32) and the Psychometric Hepatic Encephalopathy Score (PHES). HE Group (*n* = 31): This group encompassed patients with both covert/minimal HE (MHE) and overt HE (OHE), fulfilling the criteria for Type C HE (33). Specifically, patients were included if they had a prior history of OHE, were clinically assessed as West Haven grade 1–2, or were assessed as West Haven grade 0 but exhibited abnormal PHES results (indicating MHE). NHE Group (*n* = 30): This group consisted of cirrhotic patients without concurrent HE, defined strictly as West Haven grade 0 with normal PHES results and no prior history of HE episodes. HC Group (*n* = 30): Healthy volunteers with no history of systemic, psychiatric, or neurological diseases, nor any history of hypertension, diabetes, or hepatic or renal diseases. A uniform set of exclusion criteria was applied to all participants, which included: a history of alcohol consumption within the past 6 months; current use of psychoactive medications (e.g., antidepressants, neuroleptics, or benzodiazepines); age under 18 or over 75 years; and contraindications for magnetic resonance imaging (MRI) examination (such as patients with metallic implants). Furthermore, additional exclusion criteria were specifically set for patients in the HE and NHE groups: the presence of severe cardiopulmonary disease precluding study participation; concurrent hepatic malignancy or rapidly progressive liver failure; and patients with HE at West Haven grades 3–4 at the time of enrollment. This study was approved by the Biomedical Ethics Committee of the 900th Hospital of the Joint Logistics Support Force (Ethics Committee Approval No. 2025-120). All participants provided written informed consent prior to the commencement of the study.

### 2.2 Neuropsychological tests

All participants underwent the Mini-Mental State Examination (MMSE), the Montreal Cognitive Assessment (MoCA), and the PHES. All assessments were conducted on the same day as the MRI examination, in a quiet, well-lit room. The results of the PHES were adjusted according to the age- and education-level-corrected normative standards established for the Chinese population (34).

### 2.3 Laboratory examinations

To comprehensively evaluate the condition of cirrhotic patients, peripheral blood samples were collected from these subjects 3 days prior to the cranial MRI examination. The following laboratory parameters were measured: Alanine Aminotransferase (ALT), Aspartate Aminotransferase (AST), Albumin, Cholinesterase, Prothrombin Time (PT), International Normalized Ratio (INR), Serum Total Bilirubin, Creatinine, Estimated Glomerular Filtration Rate (eGFR), Serum Sodium, Blood Ammonia, Red Blood Cell Count, White Blood Cell Count, Platelet Count, and Hemoglobin. Furthermore, the Child-Pugh score was employed to assess the severity and prognosis of liver cirrhosis.

### 2.4 Image acquisition and preprocessing

All imaging data were acquired using a 3.0T United Imaging uMR770 superconducting MRI system equipped with a 32-channel head coil. High-resolution three-dimensional T1-weighted images (3D-T1WI) were obtained with the following parameters: repetition time (TR) = 7 ms, echo time (TE) = 3 ms, flip angle (FA) = 9°, slice thickness = 1 mm, readout field of view (FOV) = 256 mm, and phase FOV = 256 mm. All 3D-T1WI underwent fully automated cortical reconstruction using FreeSurfer (version 8.1.0). We performed rigorous quality control and necessary manual corrections on all results. This specifically included systematic visual inspection of brain tissue extraction, gray/white matter segmentation, and pial/dura boundary delineation. For identified issues such as registration errors, segmentation anomalies (e.g., in complex brain regions such as the insula and cingulate gyrus), and inadvertent inclusion of dura signal into the cortex, we conducted manual corrections using FreeSurfer’s built-in tools, primarily involving modification of the brain mask (gcut), addition of gray/white matter control points, and refinement of white matter and pial surfaces (white/pial edits), so as to ensure precise alignment of the pial surface with the cortical gray matter and avoid contamination from dura signal. Only data that passed quality control were included in subsequent analyses to ensure the reliability and accuracy of the study results.

### 2.5 MIND network architecture

- Brain region parcellation and feature extraction: To construct the MIND network, the five vertex-level morphological features, cortical thickness (CT), gray matter volume (GMV), surface area (SA), mean curvature (MC), and sulcal depth (SD), were first standardized. Given the different units and scales of these features, a Z-score normalization was applied across all vertices within each subject to ensure comparable distributions. Next, the normalized vertices were aggregated into 400 distinct multivariate distributions corresponding to the Schaefer 400 atlas parcellations (35).
- MIND calculation: To quantify the morphological similarity between any two regions (a and b), we calculated the symmetric KL divergence. To ensure numerical stability and avoid division by zero during the probability density estimation, a minimal smoothing constant was added to the distributions. See Equation 1:

$$\text{MIND}\;(a,b)=\frac{1}{1+\mathrm{KL}\;(a,b)}$$ (1)

In the formula, *a* and *b* represent two specific brain regions.

**Figure 1:**
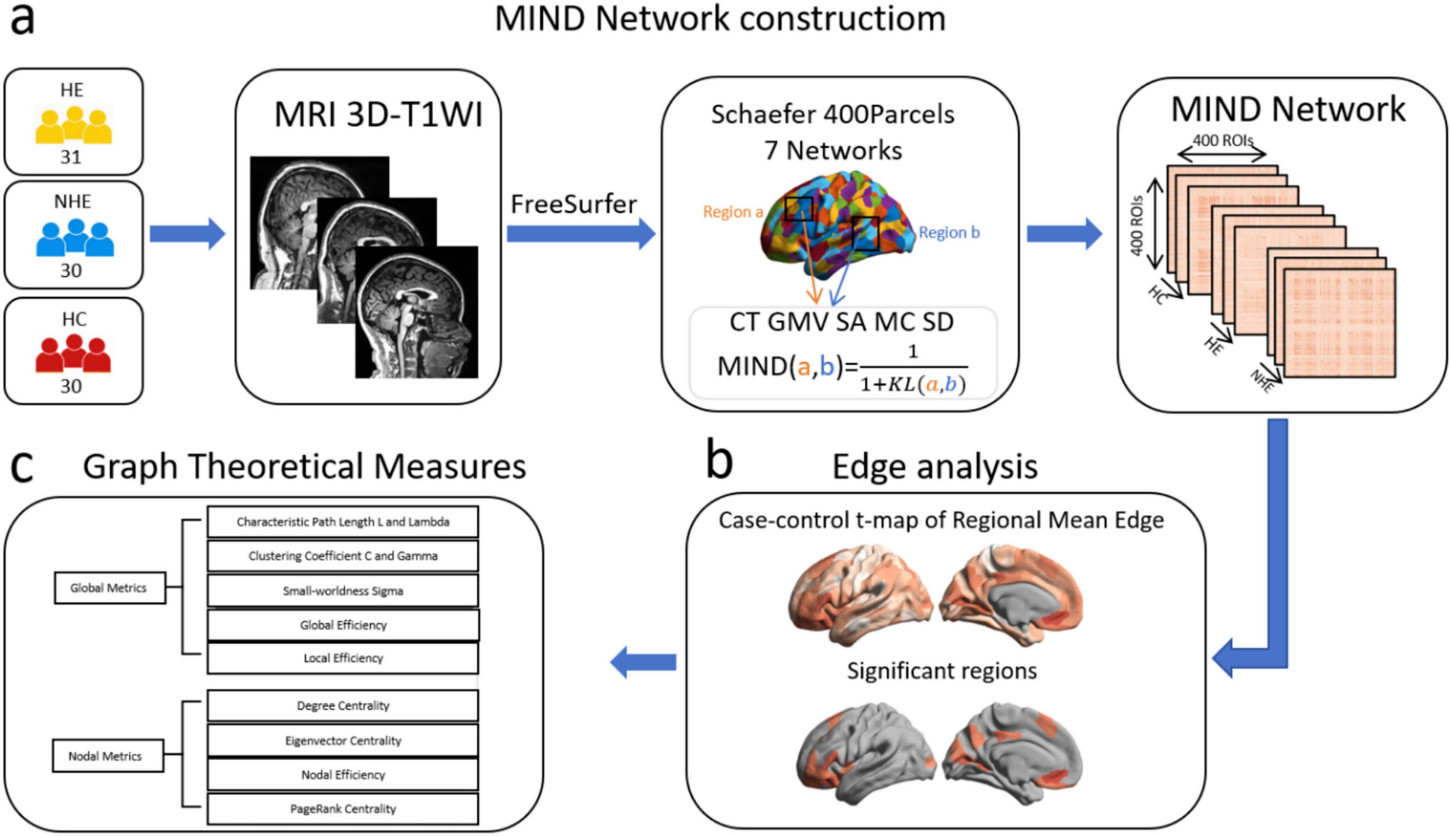
Workflow for MIND network construction and analysis. **(a)** The MIND network construction process: (1) Cortical surfaces for all subjects were reconstructed from 3D-T1WI images using FreeSurfer. (2) The Schaefer 400 atlas was mapped onto each participant’s cortical surface to parcellate it into 400 regions. For each region, five vertex-level morphological features were extracted: cortical thickness (CT), gray matter volume (GMV), surface area (SA), mean curvature (MC), and sulcal depth (SD). (3) The structural features were normalized across scales. The morphological indices from all vertices within each cortical region (as defined by the Schaefer 400 atlas) were then aggregated to form regional multivariate distributions. (4) The symmetric Kullback–Leibler (KL) divergence was estimated for each pair of regions, and a 400 × 400 MIND similarity matrix was constructed for each participant. **(b)** Group-level MIND pattern analysis was performed based on the average MIND value for each region or across all edge connections. **(c)** Further graph-theoretical analyses were conducted using global and nodal metrics. HE, Hepatic Encephalopathy; NHE, Non-Hepatic Encephalopathy; HC, Healthy Control.

Based on the aforementioned formula, the symmetric KL divergence was converted into a similarity metric, with its value range normalized to 0–1. A higher resulting MIND value indicates greater morphological similarity. Ultimately, a 400 × 400 MIND similarity matrix was constructed for each individual (as shown in Figure 1a).

### 2.6 MIND network analysis

We compared the MIND differences among the HE group, NHE group, and HC group at the level of 400 brain regions, as well as the average MIND differences across seven subnetwork regions. These seven subnetworks were defined based on the Yeo 7-network functional atlas and specifically include the Visual Network (VIS), Somatomotor Network (SMN), Dorsal Attention Network (DAN), Ventral Attention Network (VAN), Frontoparietal Control Network (FPN), Default Mode Network (DMN), and Limbic Network (LMB). Additionally, we employed edge-based fine-grained analysis to identify more subtle regional alterations.

First, to compare the overall differences in MIND networks among the three groups, we calculated the functional connection strength values (i.e., MIND values) for all 400 brain regions across the whole brain, as well as the average values of all functional connection strengths within each of the seven subnetworks (i.e., mean MIND values). A General Linear Model (GLM) was used for between-group comparisons (HE vs. NHE vs. HC), with potential confounders affecting brain function, namely sex and age, included as covariates in the model. The False Discovery Rate (FDR) method was applied to correct *p*-values for multiple comparisons to control the false positive rate (Figure 1a).

Second, to detect differences in connection patterns between specific brain region pairs among the three groups, we conducted edge-based analysis. For all 400 nodes across the whole brain, between-group comparisons were performed for each edge, resulting in a total of 400 * 399/2 = 79,800 edges. Similarly, GLM was employed, with sex and age as covariates, to perform statistical tests among the three groups for each edge (Figure 1b).

To effectively control false positives, we applied false discovery rate (FDR) correction to the statistical results of 79,800 edges. This method generated an empirical null distribution via 10,000 permutation tests and corrected the *p*-value for each edge. Compared with the conventional parametric FDR approach, the non-parametric FDR is more robust to the non-normal distributions and arbitrary dependency structures commonly observed in neuroimaging data, thereby ensuring the reliability of the results.

### 2.7 Analysis of topological properties in graph theory

This study defined the sparsity range as follows. The lower bound of sparsity ensured that the average degree of the network was greater than logN, meaning the network sparsity exceeded logN/(*N* − 1), where *N* is the number of nodes. The upper bound ensured that the networks of all participants adhered to the small-world property, with a Sigma value greater than 1.1. The continuous MIND matrices were binarized by applying a series of sparsity thresholds ranging from 0.01 to 0.5, with a step size of 0.01, generating a series of binary networks.

For the MIND network at each sparsity level, we computed both global and nodal-level metrics (as shown in Figure 1c). Global metrics included the characteristic path length (Lp), clustering coefficient (Cp), normalized characteristic path length (λ), normalized clustering coefficient (γ), small-world property (σ), global efficiency (Eglob), and local efficiency (Eloc). Nodal-level metrics comprised Degree Centrality (DC), Eigenvector Centrality (EC), Nodal Efficiency (NE), and PageRank Centrality (PC).

Although the network matrices were initially binarized over a wide sparsity range (0.01–0.5) generated by the software, the actual statistical analyses for the Area Under the Curve (AUC) of topological metrics were restricted to a rigorously defined range of 0.10–0.34. Subsequently, for each metric, we calculated the AUC within this specific sparsity range (0.10–0.34) and performed between-group comparisons. This AUC metric provides a summarized scalar value across multiple sparsity levels, offering higher sensitivity. We also established a GLM and conducted group *t*-tests with age and sex as covariates. The FDR correction was applied to adjust for multiple comparisons at the nodal level.

## 3 Results

### 3.1 Demographic and clinical data

As shown in Table 1, this study ultimately enrolled 31 participants in the HE group, 30 in the NHE group, and 30 in the HC group. No statistically significant differences were observed among the three groups regarding age, sex, or education level (*p* > 0.05). Compared to both the NHE and HC groups, the HE group demonstrated significantly lower scores on the MMSE, MoCA, and PHES (*p* < 0.001). Furthermore, the HE group exhibited significantly higher blood ammonia levels than the NHE group (*p* < 0.001), a significantly higher Child-Pugh score (*p* = 0.001), and significantly elevated total bilirubin levels (*p* = 0.030). Conversely, the HE group showed significantly lower levels of cholinesterase (*p* = 0.010) and platelet count (*p* = 0.003), along with a significantly prolonged PT (*p* = 0.002) and a significantly increased INR (*p* = 0.003) compared to the NHE group. No statistically significant difference was found in hemoglobin levels between the two groups (*p* = 0.572). The remaining clinical indicators showed no significant differences between the groups (*p* > 0.05).

**Table 1:** Demographic, clinical, and laboratory characteristics of the HE, NHE and HC groups.

Mean ± SD/median	HE (*n* = 31)	NHE (*n* = 30)	HC (*n* = 30)	Statistic	*P*-value
Gender (male/female)	23/8	15/15	17/13	3.999	0.135
Age, mean ± sd	58.81 ± 8.42	54.20 ± 9.02	55.90 ± 9.70	2.023	0.138
Education, mean ± sd	7.81 ± 2.61	8.27 ± 2.93	8.00 ± 3.12	0.194	0.824
MMSE, median (IQR)	27.00 (26.00, 28.50)	28.00 (27.00, 30.00)	30.00 (30.00, 30.00)	33.346	<0.001^a^
MoCA, median (IQR)	25.00 (23.00, 27.50)	28.00 (27.00, 29.75)	30.00 (29.00, 30.00)	30.616	<0.001^b^
PHES, median (IQR)	−4.00 (−6.00, −2.50)	−1.00 (−2.00, 0.00)	0.00 (−1.00, 0.00)	34.109	<0.001^b^
Ammonia, mean ± sd	100.02 ± 40.49	58.52 ± 36.57	NA	4.196	<0.001
Sodium, mean ± sd	141.30 (137.20, 142.50)	140.50 (138.48, 142.82)	NA	440.000	0.724
Creatinine (umol/L), median (IQR)	68.30 (61.00, 87.85)	69.00 (58.15, 79.40)	NA	532.500	0.334
Albumin (g/L), mean ± sd	31.86 ± 5.26	34.10 ± 6.01	NA	−1.547	0.127
Total bilirubin (umol/L), median (IQR)	40.60 (24.45, 67.85)	22.55 (14.45, 45.62)	NA	616.000	0.030
ALT (U/L), median (IQR)	25.20 (16.00, 33.20)	28.65 (21.88, 44.25)	NA	373.500	0.189
AST (U/L), median (IQR)	37.50 (25.50, 50.00)	40.50 (27.17, 57.75)	NA	427.500	0.593
Cholinesterase (U/L), median (IQR)	2489.20 (2036.55, 3818.05)	3878.75 (3040.57, 5568.50)	NA	286.500	0.010
eGFR (ml/min), median (IQR)	96.10 (77.74, 102.38)	99.54 (92.01, 109.48)	NA	357.000	0.121
Leukocyte (10^9^), mean ± sd	4.46 ± 1.78	4.66 ± 1.57	NA	−0.475	0.636
Erythrocyte (10^12^), mean ± sd	3.40 ± 0.90	3.66 ± 0.78	NA	−1.225	0.226
Hemoglobin (g/L), mean ± sd	107.84 ± 29.68	112.00 ± 27.37	NA	−0.569	0.572
Platelet (10^9^), median (IQR)	72.00 (55.00, 113.00)	123.50 (82.50, 166.00)	NA	257.000	0.003
PT (sec), mean ± sd	14.70 (13.90, 16.10)	13.20 (12.80, 14.67)	NA	676.000	0.002
INR, mean ± sd	1.36 ± 0.21	1.20 ± 0.18	NA	3.088	0.003
Child-Pugh scores, median (IQR)	9.00 (7.50, 10.00)	7.00 (6.00, 8.00)	NA	702.500	0.001

HE, Hepatic Encephalopathy; NHE, Non-Hepatic Encephalopathy; HC, Healthy Control; sd, Standard Deviation; IQR, Interquartile Range; MMSE, Mini-Mental State Examination; MoCA, Montreal Cognitive Assessment; PHES, Psychometric Hepatic Encephalopathy Score; NA, Not Applicable/Not Available; ALT, Alanine Aminotransferase; AST, Aspartate Aminotransferase; eGFR, estimated Glomerular Filtration Rate; PT, Prothrombin Time; INR, International Normalized Ratio.

a Pairwise comparisons show significant differences between HE and NHE, HE and HC, and NHE and HC.

b Pairwise comparisons show significant differences between HE and NHE, and HE and HC.

### 3.2 MIND network anomaly

**Figure 2:**
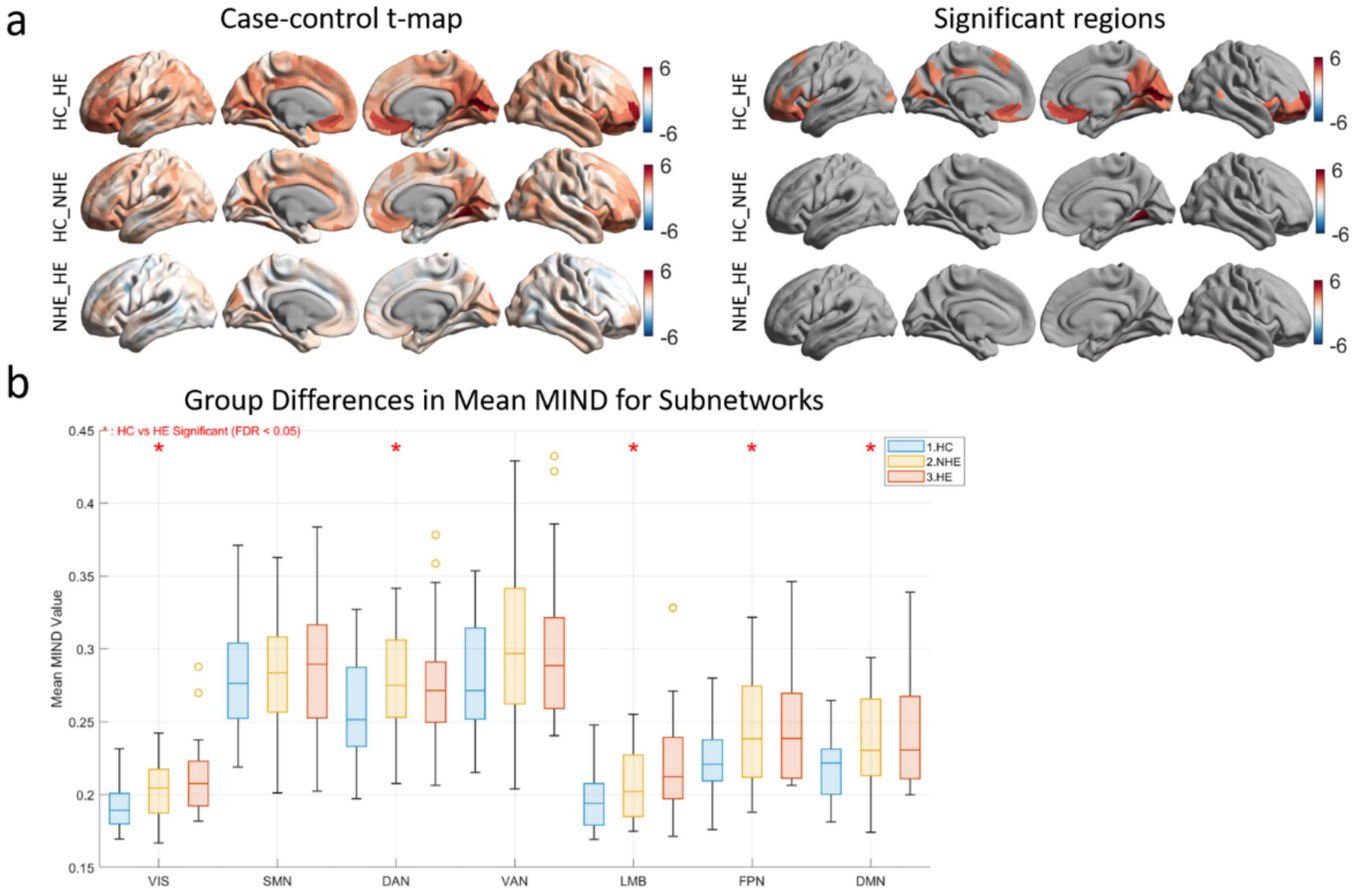
Abnormalities in the MIND network. **(a)** Case–control t-map and significant regions. Panel **(a)** displays the case–control t-maps and significant regions for the two group comparisons (HC vs. HE, NHE vs. HE). Each row represents a specific contrast, and each column represents a different viewing angle or brain slice. The color bar indicates the distribution of *t*-values, with red and orange representing higher *t*-values and blue and white representing lower *t*-values. The significant region map further highlights brain areas with statistically significant differences in the two group comparisons. **(b)** Mean MIND differences across subnetworks. Panel **(b)** illustrates the differences in mean MIND values across various subnetworks (VIS, SMN, DAN, VAN, LMB, FPN, DMN) among the HC, NHE, and HE groups. Each box plot represents a subnetwork, with the median line inside the box indicating the group’s mean MIND value, the box representing the interquartile range, and the dots indicating outliers. Asterisks (*) denote statistically significant differences between HC and HE (FDR < 0.05).

Among the MIND values calculated across 400 whole-brain regions after FDR correction (as shown in Figure 2a), compared to the HC group, the HE group exhibited significantly increased MIND values in 15 brain regions within the VIS, 6 regions within the VAN, 7 regions within the LMB, 9 regions within the FPN, and 20 regions within the DMN. In contrast, compared to the HC group, the NHE group showed a significant increase in MIND values in only 1 brain region within the VIS. No brain regions with significant MIND value alterations were found between the HE and NHE groups.

Among the mean MIND values calculated across seven subnetworks after FDR correction (as shown in Figure 2b), compared to the HC group, the MIND network of the HE group exhibited significantly higher similarity in multiple networks. This was reflected in the VIS (*t* = 3.629, *p* = 0.004), VAN (*t* = 2.212, *p* = 0.043), LMB (*t* = 2.969, *p* = 0.009), FPN (*t* = 2.917, *p* = 0.009), and DMN (*t* = 3.115, *p* = 0.009). Furthermore, no statistically significant differences were found in the comparisons between the HC and NHE groups, nor between the HE and NHE groups.

### 3.3 Edge analysis of MIND network

**Figure 3:**
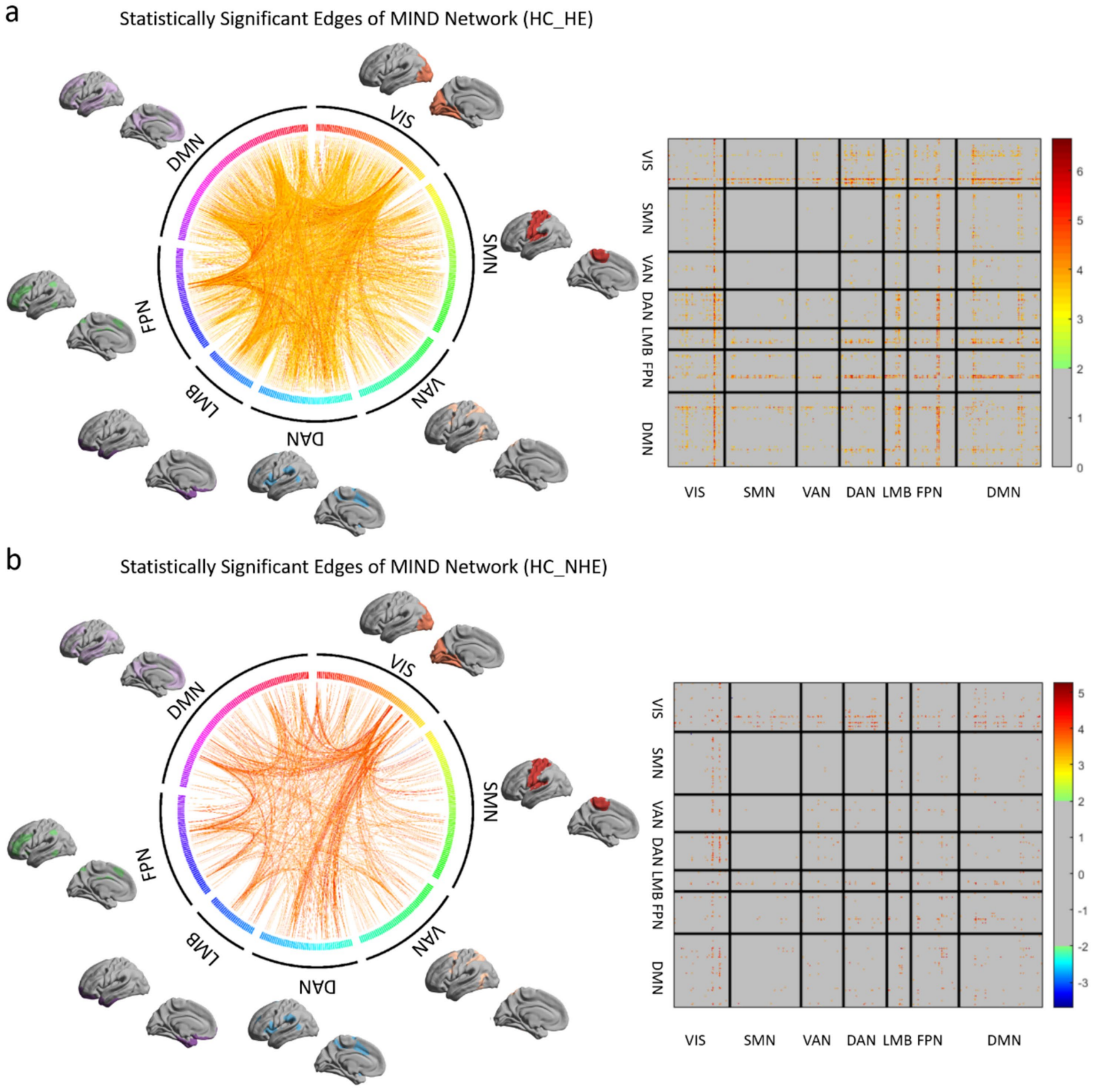
Edge analysis results of the MIND network. **(a)** Statistically significant edges of the MIND network (HC < HE). Left circular visualization: This section presents the statistically significant edges in the MIND network for the HE group compared to the HC group. The circular diagram is segmented according to different functional networks, including the DMN, VIS, SMN, DAN, VAN, LMB, and FPN. The lines represent the connecting edges within the MIND network, with different colors potentially corresponding to different types or strengths of connections. The dense central area reflects the complex interconnectivity within the network. Additionally, a three-dimensional structural schematic of the brain is included to intuitively illustrate the approximate anatomical locations of these connections. Right matrix plot: This matrix plot further quantifies the distribution of the aforementioned significant edges between different brain regions (also labeled as VIS, SMN, DAN, VAN, LMB, FPN, DMN) in a heatmap format. The color bar represents the magnitude of the statistical measure, with a gradient from blue to red indicating an increasing level of significance. This matrix allows for clear identification of which inter-regional connections exhibit significant differences between the HE and HC groups. **(b)** Statistically significant edges of the MIND network (HC < NHE). Left circular visualization: This displays the statistically significant edges in the MIND network for the NHE group compared to the HC group. It employs a similar circular structure segmented by functional network, maintaining a layout consistent with subfigure **(a)** to facilitate inter-group comparative analysis. The function of the lines and the brain schematic is identical to that in subfigure **(a)**, used to present the distribution of edges and their approximate brain locations. Right matrix plot: This matrix plot, in heatmap form, illustrates the distribution of significant edges between the NHE and HC groups across different brain regions (labeled as VIS, SMN, DAN, VAN, LMB, FPN, DMN). The meaning of the color bar is similar to subfigure **(a)**, allowing for intuitive observation of the degree and pattern of connectivity differences between the NHE and HC groups across brain regions through color variation.

After FDR correction, edge analysis of the MIND network (as shown in Figure 3) revealed that, compared with the HC group, the HE group exhibited 3,242 edges with increased network connectivity and 26 distinct patterns of increased connectivity, comprising 6 intra-network and 20 inter-network enhancement patterns. Intra-network enhancement patterns were observed within the DMN (426 edges), VAN (160 edges), SMN (94 edges), DAN (84 edges), VIS (38 edges), and FPN (18 edges), with the most pronounced increase occurring within the DMN. Inter-network enhancement patterns were prominently represented in SMN–DMN (291 edges), VAN–DMN (286 edges), DAN–DMN (250 edges), VIS–DMN (173 edges), and FPN–DMN (146 edges), with DMN-related inter-network connectivity showing the greatest prominence.

In comparison with the HC group, the NHE group displayed 732 edges with increased network connectivity and 26 distinct patterns of increased connectivity, including 6 intra-network and 20 inter-network enhancement patterns. Intra-network enhancement patterns were observed within the DMN (58 edges), VAN (56 edges), SMN (26 edges), VIS (18 edges), DAN (10 edges), and FPN (6 edges), with the most pronounced increase occurring within the DMN. Inter-network enhancement patterns were prominently represented in VAN–DMN (66 edges), SMN–DMN (65 edges), DAN–DMN (51 edges), and VIS–DMN (25 edges), with DMN-related inter-network connectivity again showing the greatest prominence. Reduced connectivity patterns included only VIS (2 edges). The above results represent the principal findings rather than an exhaustive list of all edge analysis outcomes. The HE group exhibited a greater number of edges with increased network connectivity and more pronounced enhancement effects.

In exploratory uncorrected analyses (*p* < 0.01), subtle patterns of decreased connectivity were also observed in the HE group, although these did not survive the rigorous non-parametric FDR correction. To further validate the robustness of the hyperconnectivity findings, a Network Based Statistic (NBS) sensitivity analysis was conducted. The NBS results were highly consistent with the primary FDR-corrected edge analysis, confirming the presence of a significantly enhanced subnetwork in HE patients, while no significant NBS subnetwork was identified in the NHE group (Supplementary Figure S1).

### 3.4 Abnormalities in graph-theoretic topological properties

**Figure 4:**
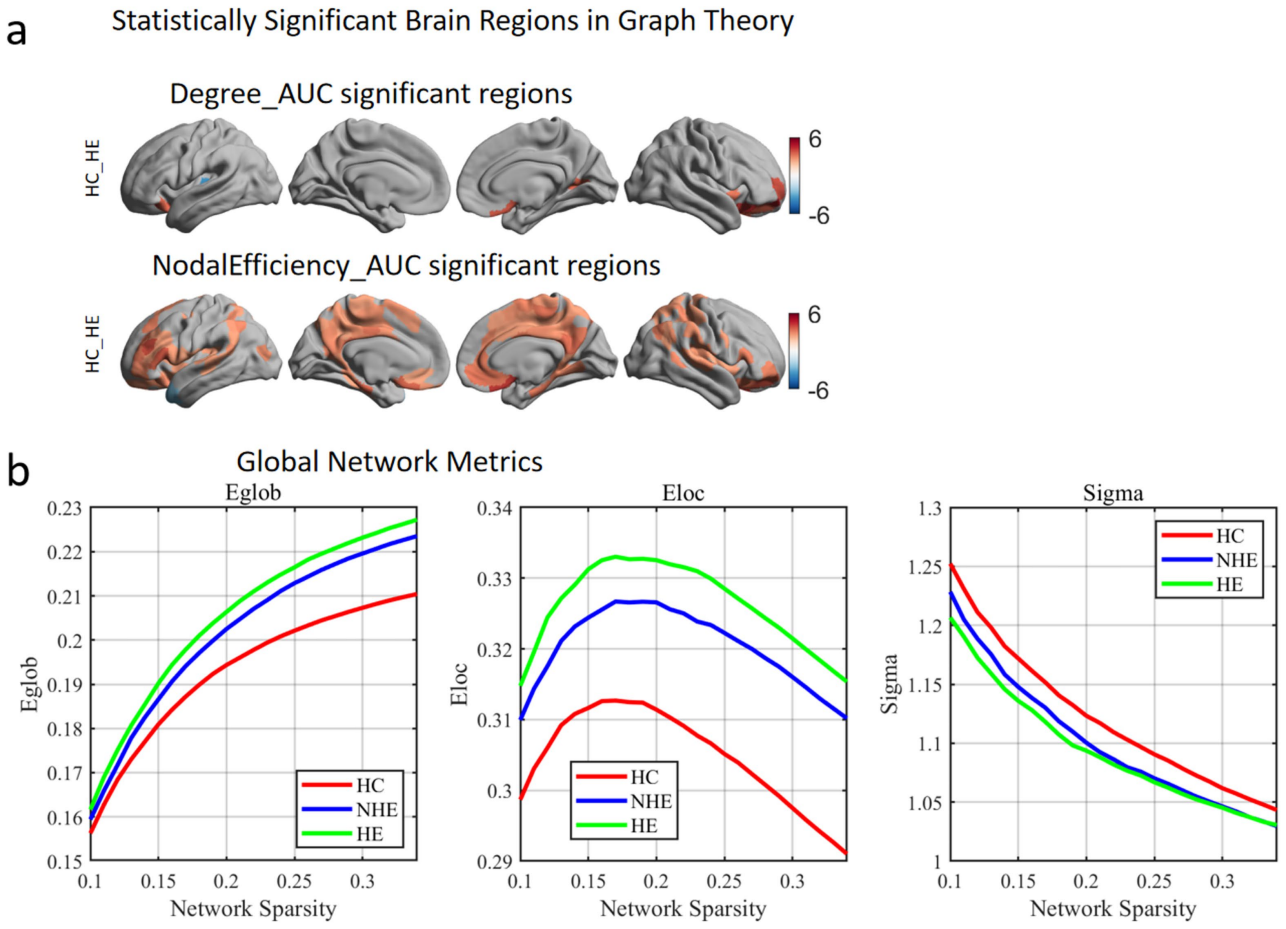
Abnormalities in graph-theoretic topological properties. **(a)** Statistically significant brain regions in graph theory. This panel displays the distribution of brain regions with statistically significant differences among the HC, NHE, and HE groups based on graph-theoretic analysis. Specifically, it illustrates significant regions for two types of nodal-level metrics: the average Area Under the Curve (AUC) for Degree Centrality (DC) and Nodal Efficiency (NE). The figure presents a three-dimensional rendered view of the brain surface, with color-coding indicating brain regions exhibiting significant inter-group differences. Red indicates regions where the metric value in the HE group is significantly higher than in the HC group, while blue indicates regions where the HE group’s value is significantly lower than that of the HC group. **(b)** Global network metrics. This panel illustrates the variations in global network metrics across different network sparsity levels (restricted to the analytical AUC range of 0.10–0.34) for the HC, NHE, and HE groups, focusing on Global Efficiency (Eglob), Local Efficiency (Eloc), and the small-world property (Sigma). The left and middle graphs show the change curves of Eglob and Eloc, respectively. Eglob is a crucial metric for measuring the efficiency of information transmission across the brain network, while Eloc reflects the information processing capacity among local clusters. The rightmost graph depicts the change curves of Sigma.

To confirm the topological validity of the selected sparsity range, the mean degree and small-worldness (σ) values at the analytical lower (0.10) and upper (0.34) bounds for all three groups are reported in Supplementary Table S1. The mean degrees at the lower bound strictly satisfy the connectedness condition, and all networks maintained small-world properties (σ > 1). Regarding nodal-level metrics (as shown in Figure 4a), compared to HC individuals, HE individuals exhibited significantly increased DC in 1 brain region within the VIS; significantly decreased DC in 1 region within the SMN; significantly increased DC in 1 region within the VAN; significantly increased DC in 4 regions within the LMB; significantly increased DC in 4 regions within the DMN; and significantly increased DC in 2 regions within the FPN. Furthermore, HE individuals showed significantly increased NE in 9 regions within the VIS, 31 regions within the SMN, 22 regions within the VAN, 10 regions within the LMB (with 1 region showing significantly decreased NE), 17 regions within the DAN, 40 regions within the DMN, and 22 regions within the FPN. No significant differences were found for the remaining nodal metrics. Additionally, no statistically significant differences were detected in the comparisons between the HC and NHE groups, nor between the HE and NHE groups.

For global-level metrics (as shown in Figure 4b), we found that the AUC for Eglob across the sparsity range (0.10 ~ 0.34) was 0.043 in the HE group, which was significantly higher than the 0.040 in the HC group (*t* = 2.681, *p* = 0.010). Similarly, the AUC for Eloc was 0.070 in the HE group, significantly higher than the 0.066 in the HC group (*t* = 2.683, *p* = 0.010). No significant differences were observed in other global metrics. It is noteworthy that, although the differences between the HC and NHE groups and between the HE and NHE groups for these global metrics were not statistically significant, Figure 4b suggests a trend where the Eglob and Eloc values for the NHE group lie between those of the HE and HC groups.

## 4 Discussion

This study is the first to integrate the MIND framework with graph-theoretic analysis in the investigation of HE, systematically revealing specific abnormalities in the cortical morphological similarity network of HE patients while delineating the transitional alteration characteristics in NHE patients. This provides a novel perspective for elucidating the neuropathological mechanisms and facilitating early diagnosis of HE. The principal findings are as follows: (1) Compared with the HC group, HE patients exhibited significantly increased mean MIND values in multiple subnetworks, with widespread intra- and inter-network connectivity enhancement, and no reduced connectivity patterns were observed; graph theoretical analysis revealed significantly elevated DC, NE, Eglob, and Eloc in multiple brain regions of HE patients. (2) NHE patients showed no significant differences in mean MIND values across subnetworks, but demonstrated a certain degree of intra- and inter-network connectivity enhancement; nodal metrics exhibited no significant differences, and efficiency indices displayed a transitional trend without reaching statistical significance.

### 4.1 Neural structural covariance mechanisms in HE revealed by MIND network

The widespread connectivity enhancement of the cortical morphological similarity network in HE patients may reflect a whole-brain systematic structural remodeling induced by chronic accumulation of neurotoxins, rather than focal injury. Such coordinated structural alterations across networks may not be merely “compensatory adaptation,” but are more likely indicative of a dedifferentiation or randomization process—namely, under pathological conditions, the brain loses its original modularity and functional specificity, and brain regions that should not co-vary synchronously exhibit excessive covariation due to diffuse morphological convergence (e.g., astrocytic swelling). This loss of network-specificity reduces the signal-to-noise ratio of information processing, leading to cognitive collapse rather than functional maintenance. The DMN plays a central role in self-referential thinking and memory retrieval (36); abnormalities in its connectivity may result in redundant and inefficient information processing (37). The enhanced intra-DMN connectivity and connectivity between the DMN and other networks (such as SMN, DAN, and VAN) in HE patients may arise because the DMN requires “deactivation” during task states; if resting-state structural connectivity is excessively enhanced, it may signify a loss of its “inhibitory function,” rendering patients unable to switch between different cognitive tasks, thereby manifesting clinically as attentional deficits and fluctuations in consciousness level. The connectivity enhancement in SMN, DAN, and VAN may be related to the neurostructural substrates of common clinical manifestations in HE patients, such as visual perceptual disturbances and attentional deficits. These widespread connectivity alterations revealed systematically by the MIND network collectively constitute the potential mechanism underlying neurostructural covariation in HE.

### 4.2 Graph-theoretic analysis reveals abnormal topological efficiency in cortical morphological networks of HE

Graph theoretical analysis further quantified abnormalities of the cortical morphological similarity network in HE patients from the perspective of network topology. At the nodal level, significantly increased DC was observed in multiple functional subnetworks (e.g., VIS, VAN, LMB, DMN, and FPN) in HE patients, with only one region exhibiting decreased DC identified in the SMN. More importantly, significantly increased NE was observed in nearly all major functional subnetworks. This indicates that in HE patients, not only the number of connections of specific brain regions within the network (i.e., DC) is altered, but more crucially, the local efficiency of these regions as network hubs for information transmission and integration is also generally enhanced.

Interestingly, while efficiency metrics (Eglob and Eloc) increased significantly, basic structural properties such as Cp and Lp remained relatively stable. Furthermore, all groups maintained their σ > 1 across the analyzed thresholds. This indicates that the HE brain does not completely degenerate into a random topology; rather, it undergoes a ‘dedifferentiation’ process. The elevated efficiency likely reflects a loss of functional segregation, rather than true structural optimization or total network randomization. However, the significantly increased Eglob and Eloc in the HE group at the global level cannot be simply interpreted as “enhanced information transmission efficiency.” In conjunction with the “dedifferentiation” theory, such “high efficiency” is more likely pathological: high Eglob implies loss of modularity and functional specificity in the brain network, whereby originally independent brain regions undergo synchronized changes due to diffuse pathological alterations, leading to redundant information processing and reduced signal-to-noise ratio; high Eloc may reflect “over-integration” of local clusters, disrupting the fine-grained nature of functional differentiation. This loss of network specificity and over-integration ultimately result in diffuse cognitive impairment (e.g., attentional deficits, executive dysfunction). Therefore, the efficiency abnormalities revealed by graph theoretical analysis are essentially a consequence of pathological network reorganization rather than a healthy compensatory state, offering a potential topological perspective for understanding cognitive deficits in HE. These MIND network indices may hold promise as quantitative markers of disease severity.

### 4.3 The transitional state of NHE and its value for early diagnosis

As a cohort of patients with liver cirrhosis but without overt HE, NHE subjects exhibited a MIND network profile characterized by “edge-level abnormalities with preserved global attributes,” providing critical clues for early warning of HE. Although NHE patients did not demonstrate statistically significant alterations in mean subnetwork connectivity strength or topological metrics, their 732 enhanced network connections spanned all major functional networks and encompassed early changes in the core aberrant networks observed in HE patients (e.g., DMN, DAN, VAN), suggesting that disruption of the cortical morphological covariance network may precede the emergence of overt cognitive symptoms.

More importantly, the network abnormality pattern in NHE patients closely resembled that of HE patients, both dominated by connectivity enhancement, differing only in the magnitude and extent of abnormalities, indicating that such network alterations in NHE represent a continuous event in the progression toward HE. Notably, Eglob and Eloc in NHE patients exhibited a transitional trend intermediate between the HE and HC groups; although statistical significance was not attained, this suggests that changes in network efficiency may commence in the early disease stage and intensify with disease progression. Therefore, the network alterations in NHE can be regarded as a continuous event in the development of HE, and the number of edge connections, connectivity strength in specific subnetworks (e.g., DMN, VAN), and the evolutionary trend of network efficiency hold promise for providing precise targets for ultra-early interventions (e.g., ammonia-lowering therapy, lifestyle modifications), thereby delaying or even preventing progression to overt HE.

### 4.4 Limitations and future directions

This study has the following limitations: first, the cross-sectional design precludes elucidation of causal relationships between MIND network abnormalities and the progression of HE, and makes it difficult to determine whether network alterations represent compensatory adaptation or pathological damage; second, the relatively limited sample size (91 cases in total across three groups) may affect detection of some weak effects. Furthermore, while clinical variables such as blood ammonia and Child-Pugh scores differ across disease stages, they were not included as covariates in our primary network analyses to avoid model overfitting and prevent over-correcting for structural variance that is intrinsically collinear with disease severity. Future studies with larger, independent cohorts are required to perform high-dimensional covariate modeling to isolate the specific impact of these metabolic markers on brain morphology. Finally, the absence of a separate MHE group prevents clarification of network abnormality characteristics at the MHE stage, and the lack of integration with multimodal data such as white matter structure and brain functional activity hinders comprehensive construction of a “structure–function” pathological model of HE.

Future research could proceed in the following directions: first, conducting longitudinal follow-up studies to dynamically observe the evolution of the MIND network during the progression of NHE patients to MHE and OHE, and to clarify the temporal characteristics of network abnormalities; second, adding an MHE subgroup to refine differences in network alterations across distinct HE stages and to screen for stage-specific biomarkers; third, integrating multimodal data such as DTI-derived white matter tractography and fMRI functional connectivity to construct an integrated “morphology-structure–function” network model, thereby deepening elucidation of the neuropathological mechanisms of HE.

## 5 Conclusion

This study, through the innovative combination of the MIND method and graph theoretical analysis, provides preliminary evidence suggesting specific abnormalities of the cortical morphological similarity network in HE patients, primarily manifested as widespread connectivity enhancement across multiple whole-brain subnetworks, increased DC and NE, and significant elevation of Eglob and Eloc. These abnormalities appear to reflect a broader, system-wide structural covariance dysregulation, which may potentially relate to a loss of network specificity due to dedifferentiation, and early edge-level abnormalities have already emerged at the NHE stage, presenting a progressive developmental pattern of “from connectivity abnormalities to topological alterations.” The findings of this study break through the traditional cognitive framework of “focal brain region injury” and provide a new interpretation of the neuropathological mechanisms of HE from the perspective of “network covariance dysregulation,” while also offering potential noninvasive neuroimaging biomarkers for early diagnosis of HE, thus holding important theoretical innovation value and clinical translational potential. With validation through larger-scale, multicenter, longitudinal studies in the future, these MIND network indices are expected to become key tools for early screening, disease monitoring, and efficacy assessment of HE.

## Data Availability

The raw data supporting the conclusions of this article will be made available by the authors, without undue reservation.
