# Supplementary material for: Investigation of cerebral cortical morphological similarity and network topological abnormalities in hepatic encephalopathy utilizing a morphometric inverse divergence network framework
Source: Front Neurol. 2026 Jul 6;17:1830519. doi: 10.3389/fneur.2026.1830519 (PMC13381429; doi:10.3389/fneur.2026.1830519)
Supplement: Supplementary file 1 [file Supplementary_file_1.docx]

| 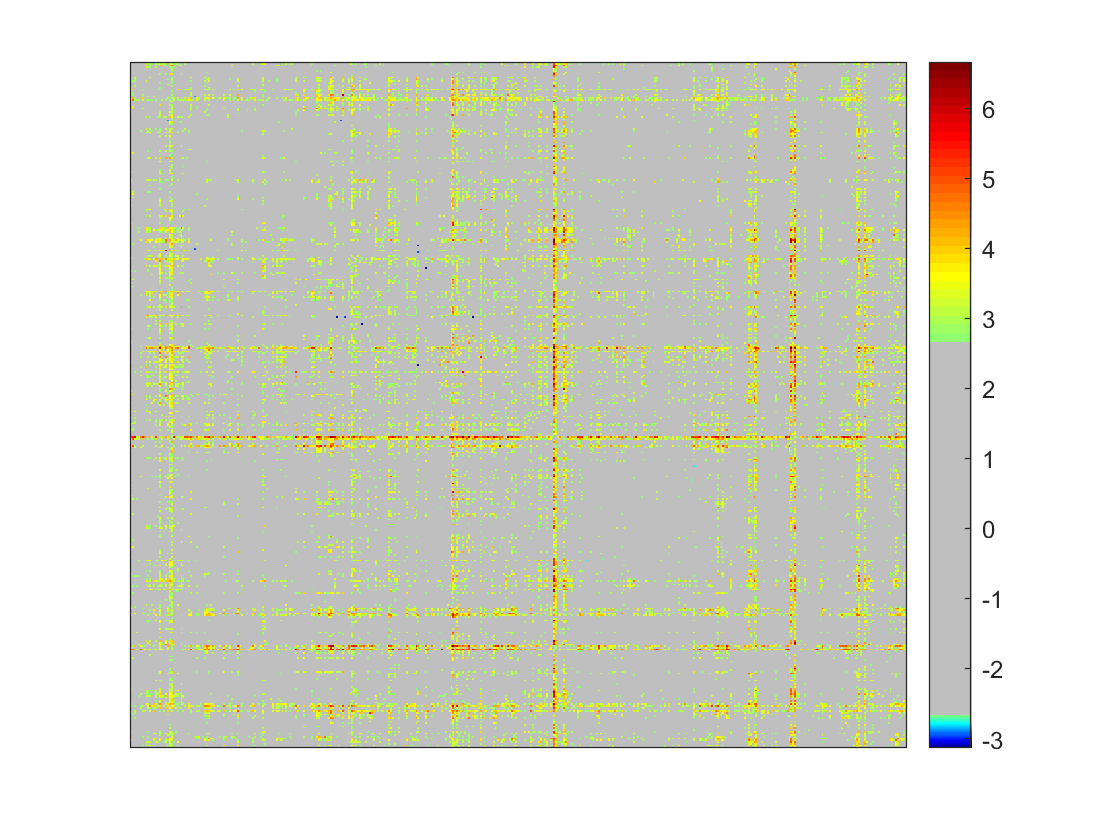  (a) | 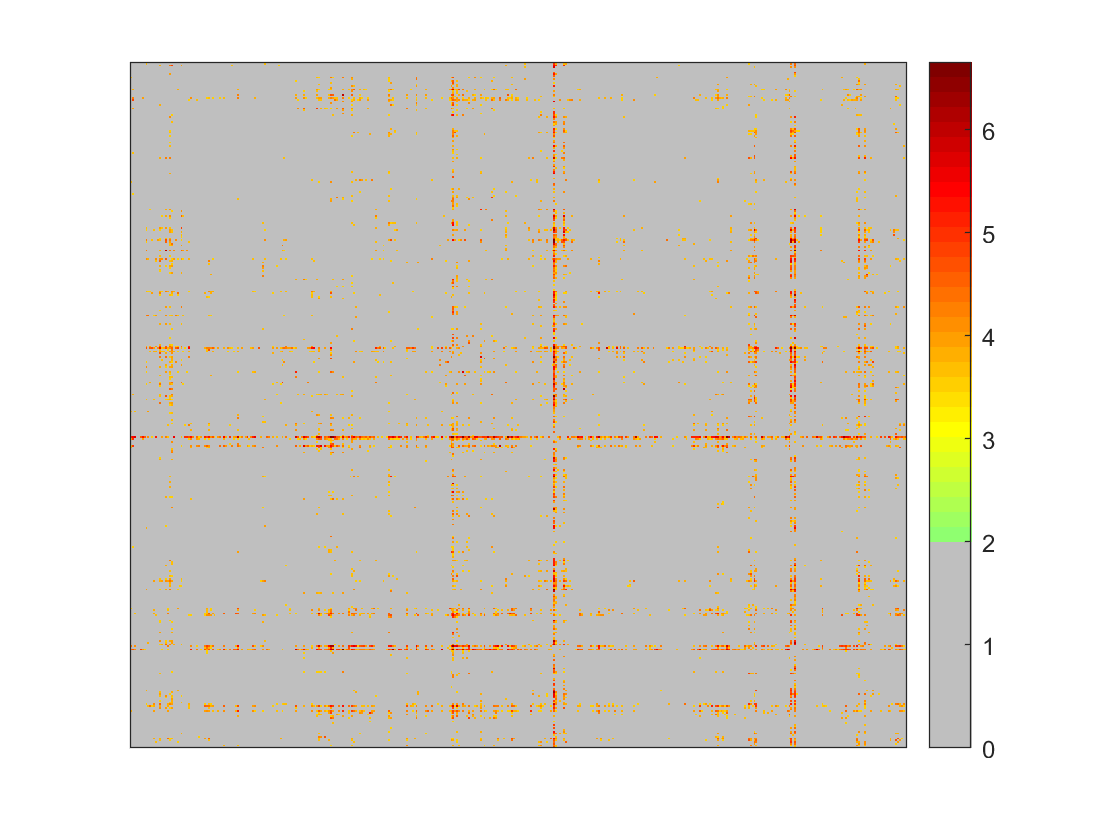  (b) |
| --- | --- |
| 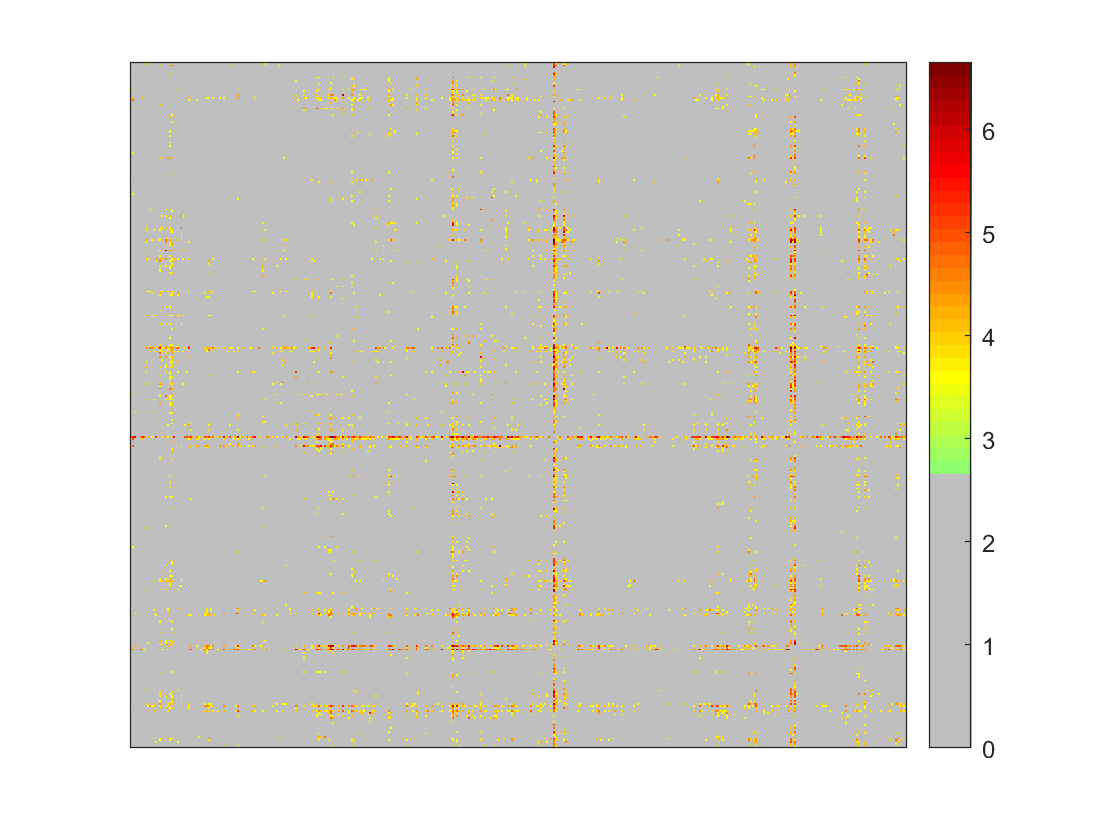  (c) | |

Figure S1: **Exploratory Edge-Level Connectivity and Network Based Statistic (NBS) Sensitivity Analysis (HE vs. HC).** (a) Exploratory uncorrected edge analysis (p < 0.01). This panel illustrates the widespread edge-level differences between the Hepatic Encephalopathy (HE) and Healthy Control (HC) groups prior to multiple comparison correction. Notably, it demonstrates the presence of both increased (warm colors) and decreased (cool colors) connections. This indicates that while subtle decreased connectivity exists in the HE brain, these signals are relatively weak and fail to survive the rigorous non-parametric False Discovery Rate (FDR) correction applied in the main analysis.(b) NBS sensitivity analysis (primary threshold p < 0.05). To validate the robustness of the hyperconnectivity pattern, an NBS approach was applied. This analysis identified a significant, large-scale interconnected subnetwork dominated by enhanced connections in the HE group, highly consistent with the primary FDR-corrected findings.(c) NBS sensitivity analysis (primary stricter threshold p < 0.01). Applying a more stringent primary threshold in the NBS framework further isolated the core hyperconnected topological hubs. The persisting subnetwork fundamentally reinforces that robust structural covariance hyperconnectivity (particularly involving the DMN) is the dominant pathological alteration in HE.Abbreviations: HE, Hepatic Encephalopathy; HC, Healthy Control; NBS, Network Based Statistic; DMN, Default Mode Network; FDR, False Discovery Rate.
