# Supplementary material for: Investigation of cerebral cortical morphological similarity and network topological abnormalities in hepatic encephalopathy utilizing a morphometric inverse divergence network framework
Source: Front Neurol. 2026 Jul 6;17:1830519. doi: 10.3389/fneur.2026.1830519 (PMC13381429; doi:10.3389/fneur.2026.1830519)
Supplement: Supplementary file 2 [file Table_1.docx]

**Table S1** Network mean degree and small-worldness (σ) at the analytical lower and upper sparsity bounds [Mean ± SD].

| Sparsity Threshold | Metric | HC (n=30) | NHE (n=30) | HE (n=31) |
| --- | --- | --- | --- | --- |
| Lower Bound (0.10) | Mean Degree | 15.845±1.733 | 16.813±2.239 | 17.065±2.243 |
|  | σ | 1.252±0.229 | 1.228±0.187 | 1.207±0.166 |
| Upper Bound (0.34) | Mean Degree | 42.366±4.461 | 45.377±6.347 | 46.131±6.676 |
|  | σ | 1.043±0.074 | 1.030±0.074 | 1.031±0.062 |

Note: At the actual analytical lower bound of 0.10, all groups exhibit a mean degree well above this threshold, ensuring network connectedness. Additionally, all groups preserve basic small-world topology (σ) across the analyzed range. Abbreviations: HC, Healthy Control; NHE, Non-Hepatic Encephalopathy; HE, Hepatic Encephalopathy; SD, Standard Deviation.
